# A quality analysis of thyroid cancer videos available on TikTok

**DOI:** 10.3389/fpubh.2023.1049728

**Published:** 2023-03-23

**Authors:** Li Wang, Yongjin Li, Jiali Gu, Li Xiao

**Affiliations:** ^1^College of Humanities, Huzhou University, Huzhou, Zhejiang, China; ^2^College of Life Sciences, Huzhou University, Huzhou, Zhejiang, China

**Keywords:** thyroid cancer, TikTok, health information, short video, social media

## Abstract

**Background:**

Thyroid cancer (TC) is becoming an increasing public health problem worldwide. TikTok is a global video-sharing social media app, which can be a source of information on TC. However, the information quality of these videos remains unknown.

**Methods:**

A search of TikTok was performed with the term “甲状腺癌”and “甲状腺肿瘤” (“thyroid cancer” and “thyroid neoplasm” in Chinese). Videos included were independently assessed using six predefined questions for content scores and DISCERN scale for information quality, while the video popularity index (VPI) was also calculated. A correlation analysis was performed among duration, presence of animation, VPI, DISCERN scores, and content scores.

**Results:**

A total of 56 videos were finally included, of which 49 were uploaded by physicians, 4 by health organizations, and 3 by hospitals. While 43 were real content videos, 13 were animated. The mean of DISCERN score and content score was 3.44 ± 0.72 and 5.19 ± 0.95, respectively. Good consistency was found between the two reviewers in terms of both DISCERN scores and content scores. The video duration and presence of animation were positively correlated with VPI, while DISCERN scores and content scores were not correlated with VPI.

**Conclusion:**

The overall quality of TC-related videos was satisfactory, although the quality varies greatly depending on the type of source. Patients should be cautious when using TikTok as a source of TC-related information.

## Introduction

Thyroid cancer is the most common malignancy of the endocrine system, with the differentiated thyroid cancers papillary and follicular accounting for the majority of new cases (1). Furthermore, TC is more likely to occur in women with an incidence of 2.5 times (2), and the five-year survival rate is 98.1% (3). An apparent increase in TC incidence was shown in recent studies (4, 5), which may be driven by a combination of increased surveillance, lifestyle, and environmental factors (6–8).

Due to an increase in health awareness, people have a greater demand for health information, especially for patients suffering from cancer. Patients are increasingly demanding to be involved in decision-making process (9). Health information could be provided by diverse sources besides professional physicians. Although such information is not a substitute for medical advice, it could reinforce knowledge and help patients to make personal decisions.

Social media is an important way to disseminate information. However, social media is also a major source of misinformation, especially health relevant information. Misinformation tends to spread faster and more broadly than verified information due to the diversity of users and lack of monitoring and censorship mechanisms. Several studies have shown the potential for social media to be misleading in disseminating health information (10–16). TikTok is a short video-sharing app that allows users to create and share videos on any topic. In China, TikTok reached the first position on the global mobile app download list in December 2021 (17). Compared with other social media, TikTok has become more popular because it is easy to produce and share short videos, including elements like music, animation, and various visual effects (18). TikTok is expanding its coverage of topics on many aspects of daily life, such as beauty and makeup, education, cooking, wellness and technology (19). Educational healthcare content has also become an important part of TikTok’s content ecosystem (20). However, the unregulated content of TikTok video and the lack of peer review process increases the likelihood of dissemination of inaccurate and large volumes of information with varying quality and credibility, raising a significant challenge in the provision of optimal healthcare. Given the increasing use of TikTok app by increasingly patients, physicians, and researchers as an effective channel for healthcare communication in recent years (21, 22), it thus is vital for patients and health care providers to understand the quality of information that patients are likely to find, and for health educators to make higher quality short video available.

Although TC is a disease with a good prognosis, receiving a cancer diagnosis is a sensational event for the general person. Correct dissemination of TC-related health information plays an important role in enhancing patient belief and maintaining good patient–physician relationships. However, to our knowledge, the quality of short video content regarding to TC has not yet been analyzed. To address this gap, this study aims to assess the quality of the short videos about TC from TikTok.

## Methods

### Search strategy

Keywords “甲状腺癌” and “甲状腺肿瘤” (“Thyroid cancer” and “thyroid neoplasm” in Chinese) was searched within TikTok app on March 20, 2022. The first 100 videos that appeared on each search were reviewed. A total of 56 videos were finally included for analyzing in this study after excluding videos with advertising content (*n* = 8), duplicated (*n* = 36), as shown in the Supplementary Figure S1.

### Data collection

All selected videos were categorized into three groups according to source: physicians, hospital channel (non-profit organization), and health organizations (for-profit organizations). In order to confirm the authenticity of the included videos, we not only carefully checked the registration information of the video uploader’s account, but also accessed the official website of the hospital where the video author worked to confirm the author’s real identity. Information of videos was extracted and coded, including source of video, the content of the videos, the presence of animation, duration (in seconds), the upload date, and other viewer interactive quality markers including number of views, likes, and comments. The VPI was calculated using the formula “(number of likes/number of views) × 100)” (12).

### Quality assessment of video information

Videos were assessed from two aspects: the quality of information and their contents. The quality of information was rated using an adapted DISCERN tool by Singh et al. (23) considering the following reasons: (1) It is a widely used tool for evaluating the quality of health information (24, 25); (2) It is useful for assessing information quality on other video-based platforms (e.g., YouTube) (26). This DISCERN tool has five questions in total, and each question is answered yes or no. Yes answer is 1 point and no answer is 0, and a maximum of 5 points can be obtained. DISCERN scores thus ranged from 1 = unacceptable, 2 = poor, 3 = acceptable, 4 = good, to 5 = excellent, higher scores indicate a greater video reliability (25). Questions used in the DISCERN scale are shown in Table 1.

**Table 1:** Assessment of reliability of videos on TC found on TikTok APP.

No.	Questions
1	Are the targets clear enough?
2	Are reliable sources of information used? (Doctors, health channel……)
3	Is the information presented balanced and unbiased?
4	Are the alternative sources offered to patients?
5	Are areas of uncertainty mentioned?

Content score was rated in terms of six predefined questions (definition of a disease, risk factors, evaluation, signs and symptoms, management, and outcomes; one point is given for covering 1–2 aspects, two points is for 3 aspects, three points is for 4 aspects, four points is for 5 aspects and five points is for full coverage. The rating criteria are the same as DISCERN scores). To facilitate statistical analysis, we recorded the basic information of each video (publication date; duration; views, likes, comments, shares; presence of animation or not) and basic information of the video publisher (account name, self-description, publisher identity). Two reviewers independently appraised the quality of the included videos.

### Ethics statement

This study focused on the quality assessment of TikTok videos contributed and viewed by the public, so ethics committee approval was not required.

### Statistical analysis

Statistical analysis was made using IBM SPSS Statistics for Windows, version 19.0. The statistical analysis methods used include descriptive statistical analysis, Pearson correlation analysis, and one-way ANOVA. The continuous variables were expressed as mean ± standard deviation, median (min-max), while nominal variables were given as frequency and percentage. *p* < 0.05 was considered statistically significant.

## Results

### Video characteristics

Of the 56 videos included in this study, 49 (87.5%) were uploaded by physicians, 4 (7.1%) by health organizations, and 3 (5.4%) by hospital channels. There wasn’t video from patients or their relatives. Forty three (76.8%) videos were real content videos, 13 (23.2%) were videos with animation.

### Descriptive statistics for videos

The duration of the videos varied from 5 to 111 s. The least online days of video were 2 days prior to data collection, whereas the most online days were nearly 3 years. Median (min-max) number of views, likes, comments, collection, sharing, and VPI were 46,211 (578–11,088,000), 1378.5 (22–308,000), 83 (0–73,000), 82.5 (1–1775), 130.5 (15–5,796) and 2.5 (2–8.82) prior to data collection. There were no significant differences among different sources of video (physician, hospital channel, and health organization) regarding such video characteristics mentioned above except for VPI between health organization and hospital channel (*p* = 0.002), physicians (*p* < 0.001).

### Quality assessment of video

**Figure 1:**
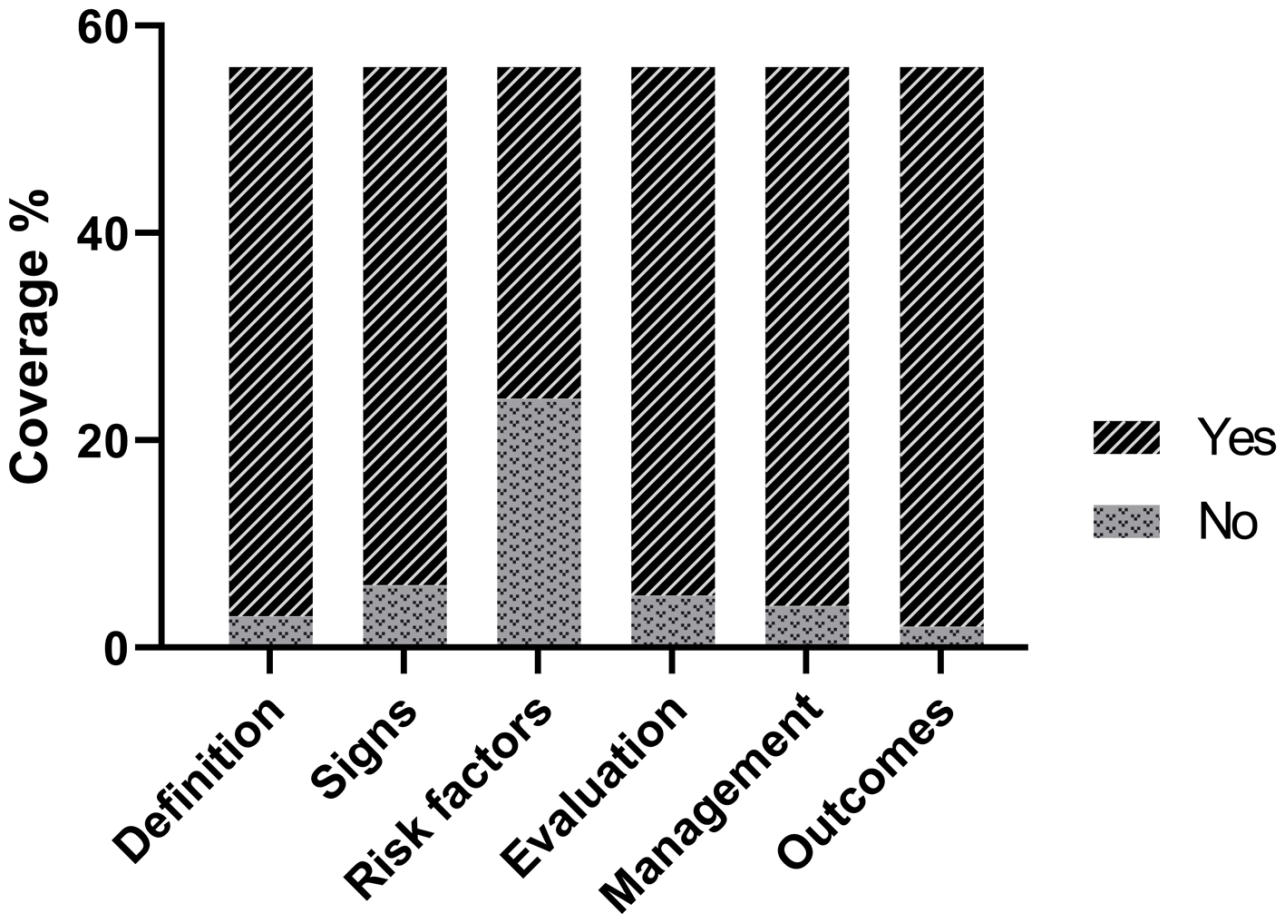
Percentage of videos addressing each TC topic.

The average DISCERN score given by the two reviewers was 3.44 ± 0.72, while the DISCERN score for individual reviewers was 3.5 ± 0.71 and 3.38 ± 0.73, respectively. A good agreement was observed, *κ* = 0.72 (*p* < 0.001). Regarding the source of videos, the videos contributed by hospital (4.0 ± 0.12) and physicians (3.4 ± 0.7) had higher DISCERN scores than the one by the health organization (2.3 ± 0.5). Significant quality difference was observed between health organizations and hospitals (*p* = 0.014), physicians (*p* = 0.006). More than 85% of videos have a DISCERN score greater than 3 (Table 2). In addition, the video content responded the predefined six questions to different degrees, as shown in Figure 1. The average content score given by the two reviewers was 5.19 ± 0.95, while for individual reviewers was 5.21 ± 0.91 and 5.16 ± 0.99, respectively. The *κ*-coefficient for content scores was 0.781 (*p* < 0.001), which indicated a good consistency between the two reviewers. The results also showed that most of videos sufficiently addressed definition (53/56, 94.6%), signs and symptoms (50/56, 89.3%), management (52/56, 92.9%), evaluation (51/56, 91.1%) and outcomes (54/56, 96.4%), while risk factors (32/56, 57.1%) were absent in 42.9% videos.

**Table 2:** General characteristics of the videos.

	Mean ± SD	Median	Min	Max
Video duration (second)	**41.45 ± 21.18**	**37**	**5**	**111**
Number of days online (days)	**243.18 ± 278.18**	**129**	**2**	**1,082**
Views (count)	**298919.16 ± 1473716.1**	**46,211**	**578**	**11,088,000**
Likes (count)	**8020.61 ± 40954.84**	**1378.5**	**22**	**308,000**
Comments (count)	**1460.54 ± 9736.43**	**83**	**0**	**73,000**
Collection (count)	**266.5 ± 403.82**	**82.5**	**1**	**1775**
Sharing (count)	**542.05 ± 1008.05**	**130.5**	**15**	**5,796**
VPI (%)	**2.68 ± 1.02**	**2.5**	**2**	**8.82**
DISCERN score reviewer 1	**3.5 ± 0.71**	**2**	**0**	**3**
reviewer 2	**3.38 ± 0.73**	**2**	**0**	**3**
Content score reviewer 1 Reviewer 2 Good quality videos	**5.21 ± 0.91** **5.16 ± 0.99** **n (%)**	**5** **5**	**3** **2**	**6** **6**
DISCERN score ≥ 3 reviewer 1	**49 (87.5)**			
reviewer 2	**48 (85.7)**			
Source of upload	**n (%)**			
Physicians	**49 (87.5)**			
Hospitals (non-profit)	**3 (5.4)**			
Health organization (for-profit)	**4 (7.1)**			
Presence of animation	**n (%)**			
Yes	**17 (30.3)**			
No	**39 (69.6)**			

The top 10 videos, the source of videos, the video content, the upload date, and VPI values are given in Table 3.

**Table 3:** General characteristics and VPI values of the top 10 videos.

Uploader source	Content	Upload date	VPI (%)
Hospital channel	**Education**	**12/01/2020**	**8.82**
Hospital channel	**Education**	**04/07/2019**	**5.75**
Physician	**Education**	**09/20/2021**	**3.33**
Physician	**Education**	**12/02/2021**	**3.33**
Physician	**Education**	**04/01/2020**	**3.33**
Physician	**Education**	**12/23/2021**	**3.23**
Physician	**Education**	**08/06/2021**	**3.13**
Physician	**Education**	**08/03/2021**	**3.13**
Physician	**Education**	**03/05/2022**	**3.03**
Physician	**Education**	**01/20/2022**	**3.03**

### Correlations between descriptive parameters

**Figure 2:**
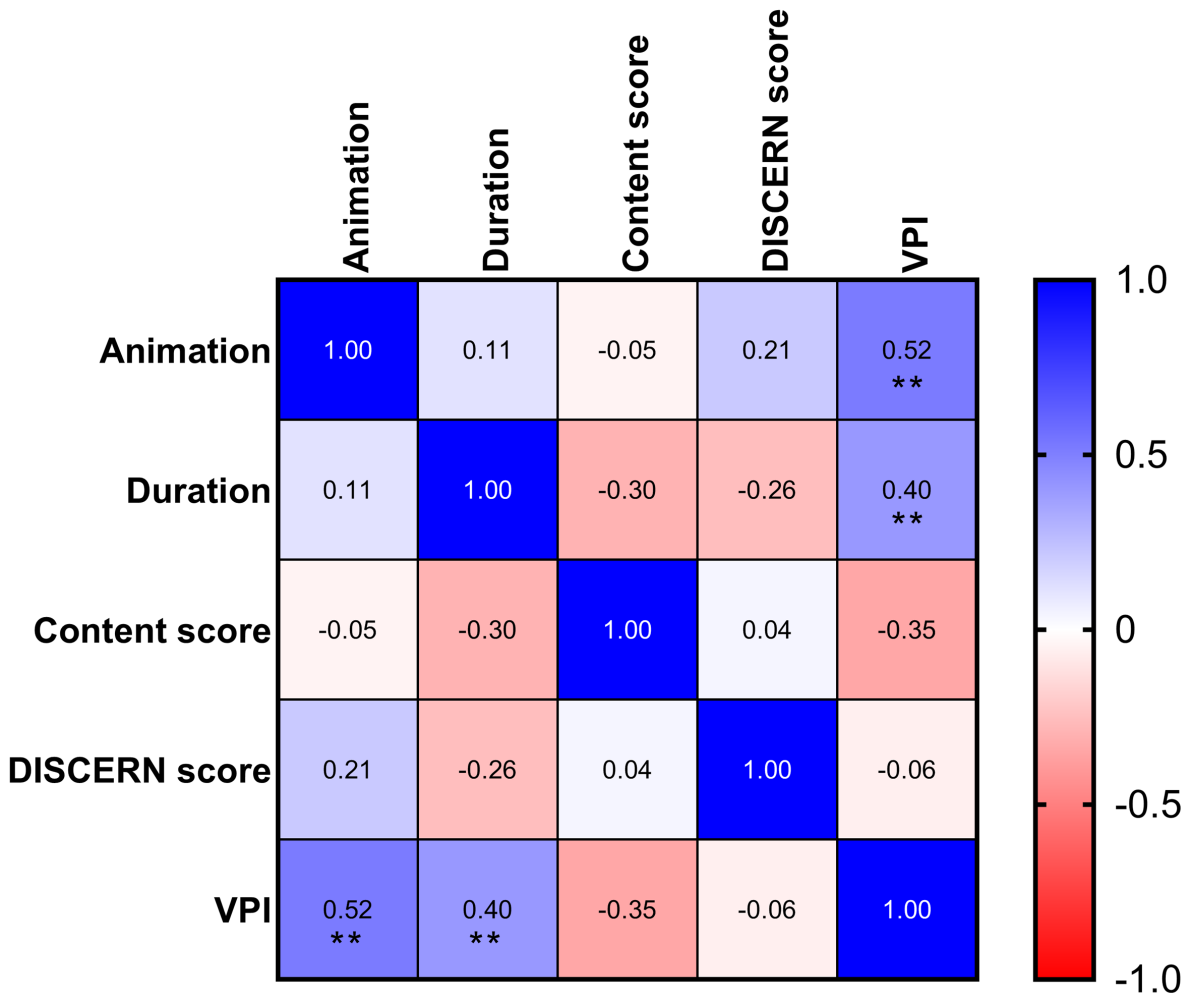
Correlations considering animation, duration, VPI, DISCERN scores, and content scores. **p < 0.01.

The duration of the video and presence of animation were positively correlated with VPI of videos (duration: *r* = 0.40, *p* = 0.002; animation: *r* = 0.52, *p* < 0.001). Both DISCERN score and content score were not correlated with VPI, and no correlation was noted between DISCERN score and content score, as shown in Figure 2.

## Discussion

### TikTok as information source

Video-based social media were important platforms for producing and disseminating health-related videos. Recent evidence indicates that TikTok has demonstrated strong communication potential during the COVID-19 pandemic (27), however, the role of TikTok in disseminating TC relevant information remains unclear.

Our results suggest that the overall quality of TC-related videos from TikTok was satisfactory. Videos included in this study have received approximately 16.74 million views since they were uploaded. These results indicated that TikTok is a new source of information on TC.

### Information quality appraisal

TikTok app is primarily an entertainment-oriented application which may differentiate itself from other social media by publishing quirky videos rather than serious professional content (18). As the number of users grows, some professional knowledge sharing videos, such as medical education, have also been integrated into TikTok’s content ecosystem (21, 28). Our results here indicated that the quality of video on TC found on TikTok was satisfactory. These results were consistent with some previous studies about other disease videos found on TikTok (29–31), which found that the information provided in these videos was generally reliable.

Our results also show that the videos included in this study all covered predefined relevant questions to varying degrees, which may be due to the duration limit of TikTok videos (the duration of videos for new users is limited to 15 s, and longer videos require a certain number of fans and views) (32). The most frequently mentioned topic was the outcomes of TC, which may be due to the fact that this disease is the nature of cancer and is not generally known by the general population to have a low rate of malignancy. It is clearly helpful for patients to improve their risk perception of the prognosis of the disease by using TikTok to communicate such information to them.

TikTok app has a strict audit system for video publishers of health knowledge sharing (33). Therefore, there are no videos uploaded by patients or their relatives in the first 100 videos we search each time. Of the included 56 videos, 49 (87.5%) were uploaded by physicians, 4 (7.1%) by health organizations, and 3 (5.4%) by hospital channels. Videos published by physicians and hospitals had the higher quality which have been confirmed by many previous studies, while those from the health organizations for profit had the lowest quality (34, 35). These results indicated that physicians and government-sponsored platforms are more likely to publish high-quality information than for-profit organizations. However, the videos contributed by the hospital channel account for a mere portion of the total videos on TikTok. Considering the busy routine of physicians, we therefore suggest that public hospitals organize professionals to contribute more high-quality videos to patients and leverage the power of this social media channel to promote public health.

For videos found on TikTok, the video popularity attributes to many factors, such as the rules set by TikTok and common social interactions, even the psychological effects of the audience (36), which challenge to find videos from trustworthy sources. Therefore, since trustworthiness is not the only criterion for TikTok ranking, it is possible that videos from trusted sources will receive a lower ranking, while misleading and incorrect videos may receive a higher ranking. Notably, a new algorithm is needed to enable higher user acceptance and trusted videos remain in the top positions in order to help patients to access quality and trusted video resources.

Animated videos in previous study (10) were reported to be correlated positively with the number of views and negatively with the video duration. However, videos with animation had no advantage over non-animated videos in terms of number of views, DISCERN scores, or content scores in our study. This may be due to the small number of animated videos and the use of similar and repeated animation resources. In addition, a weakly positive correlation existed between duration, animation, and VPI. This may attribute to the type of video. Unlike entertainment videos, health knowledge sharing videos need longer duration to accommodate more information. TikTok has recently raised its video duration limit to 10 min (37), which is good for health-sharing videos.

## Limitations

Limitations to this study should be mentioned. First, the search results on TikTok are dynamic over time; the results thus might vary with the use of different search dates and time. Second, the research results may vary according to the geographical location of the viewer. Third, video sampling retrieves only the first 100 in each search which can lead to inadequate coverage.

## Conclusion

In this study, we analyzed the quality of information provided by TikTok video regarding TC. The overall quality of information based on DISCERN scores and content scores was satisfactory. Our finding also supports TikTok as a new source of information on TC, and therefore, physicians and hospitals should embrace this evolving technology and provide videos with higher quality to improve patients’ awareness about TC. Patients should also be cautious when watching TC relevant videos on TikTok due to the complexity of VPI.

## Data availability statement

The original contributions presented in the study are included in the article/Supplementary material, further inquiries can be directed to the corresponding author.

## Author contributions

LW and YL: Conceptualization and Methodology. JG and LX: Experiments and Data analysis. LW and YL: Writing. All authors contributed to the article and approved the submitted version.

## Funding

This work was supported by Science and Technology Department of Zhejiang Province (LGC21H200001) and Huzhou Science and Technology Bureau (2020GZ29).

## Conflict of interest

The authors declare that the research was conducted in the absence of any commercial or financial relationships that could be construed as a potential conflict of interest.

## Publisher’s note

All claims expressed in this article are solely those of the authors and do not necessarily represent those of their affiliated organizations, or those of the publisher, the editors and the reviewers. Any product that may be evaluated in this article, or claim that may be made by its manufacturer, is not guaranteed or endorsed by the publisher.
