# Supplementary figures and images for: A quality analysis of thyroid cancer videos available on TikTok
Source: Front Public Health. 2023 Mar 23;11:1049728. doi: 10.3389/fpubh.2023.1049728 (PMC10076716; doi:10.3389/fpubh.2023.1049728)

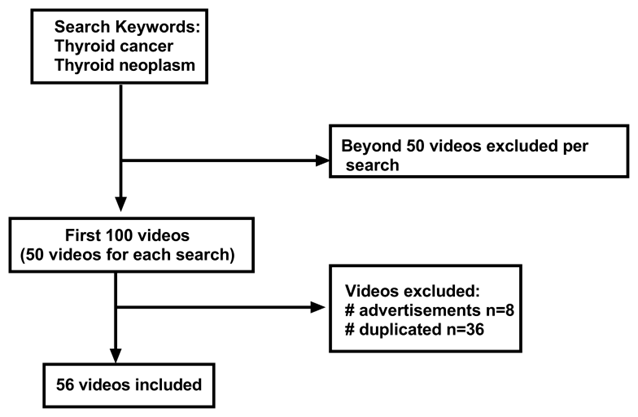


**sFig. 1 Video selection process**

Supplement: Supplementary file 1 [file Table_2.DOCX]
